# Damage control surgery–new concept or reenacting of a classical ideea?

**Published:** 2008-08-15

**Authors:** M Beuran, FM Iordache

**Affiliations:** Department of Surgery, Emergency Clinical HospitalRomania

## Abstract

Damage–control surgery is an example of a paradigm shift. The term is borrowed from naval terminology and means gaining the initial control of a damaged ship. Because of the lethal triad the polytrauma patient is at a grave risk. The classical concept of surgically solving all the patient's injuries in the first moment was even theoretically incorrect as a multiple injured patient is a critical patient with depleted reserves. As such, complex procedures were doomed from this point of view. The concept of damage–control surgery emerged in 1992. The core idea was that as minimal as possible had to be done in these critical patients in the first phase, meaning temporary control of a hemorrhage and simple measures for stopping contamination. After 24–48 hours in the ICU, in which time the physiological disturbances were corrected, a further intervention is performed for definitively treating the injuries. Further refinements consider five stages and not three in damage–control surgery. The bright side of the concept is an up to 70% survivability rate but with a higher risk of complications, mostly due to the policy of temporary closing the abdomen and sepsis.

**What is damage–control surgery?**

In turn with the high frequency of trauma on a global scale, during the last decade a new concept has been developed. This is the challenging concept of damage control–surgery. What has changed in our understanding of trauma? Why do we need this concept? To understand this ‘paradigm shift’(W. C. Schwab) we must aknowledge the trauma patient's primary characteristic which is that of a critical patient with unknown potential, in a state of major imbalance of it's vital systems and functions. The classic therapeutic concept was definitive care of all patient's injuries (early total care). This often lead to to the following asertion: ‘a patient completly fixed by surgical standards but.. dead’.

The term ‘damage–control’ originates from US Navy referring to the ability of a ship to absorb damage while maintaining mission integrity [1]. Lethal triad defined by hypothermia, hypocoagulability and metabolic acidosis can rapidly emerge in trauma patient. Its installment makes almost impossible salvaging the patient. The solution seems to be a pathophysiologically oriented approach to the problem.

The term was coined in 1992 by Rotondo et al. pleading for restoring as fast as possible normal physiology postponing definitive surgical treatment [2]. The success rate reported by various authors when applying damage–control surgery can be as high as 60% [3].

The original authors have initially suggested the three–stage approach when applying damage–control surgery.

First stage takes place in the operating room and has the following purposes: control of haemorrhage, exploration, control of contamination, definitive packing, and rapid abdominal closure.

The surgical procedure is restricted to a bare minimum. This concept of abbreviated laparotomy was first introduced by Stone et al. in 1983. In a study group of 31 patients, he had 12 of 17 patients with abbreviated laparotomy who survived. Still, the moment for stopping or limiting the surgical procedure was decided by the surgeon when he or she thought that death was imminent [4]. Stone used the term ‘bail–out surgery’, underlying the in extremis characteristics of the procedure. It is obvious that defining objective criteria represents a problem for choosing when to stop the procedure.

Abbreviated laparotomy entails a complete exploration of the abdomen. Bleeding will be controlled as fast as possible by sutures or by temporary vascular shunts where ligating the vessel would have serious repercussions (e.g. superior mesenteric artery). For solid organs, stopping the bleeding refers especially to abdominal packing (mostly the liver being involved).

Contamination control comes next and is achieved by simple means. In this dramatic situation no resections with complex anastomosis will be performed. For this purpose, staplers are invaluable, drastically reducing the procedure duration. These devices allow quick closing and removing of damaged bowel. Restoring the continuity of the digestive tract can be done at a later time. For polytrauma patients who have undergone damage–control procedures, the abdominal wall's continuity will most likely not be restorable during the initial procedure, due to visceral edema and the procedure itself (e.g. hepatic packing). Attempting this is difficult, if not impossible, and abdominal compartment syndrome would be an immediate consequence which would be potentially lethal to the patient [5]. For this reason, various methods of temporarily closing the abdomen will be used, such as towel clips, Bogota bag, vacuum–packs, Wittmann patch (a Velcro–like device), zippers, various types of meshes and others. Temporary closure of the abdomen has the following objectives:

- containing the intra–abdominal organs;
- controlling the abdominal secretions;
- maintaining haemostatic pressure;
- achieving secondary closure without further complications.

The second stage of damage–control takes place in the intensive care unit (ICU). During this stage all imbalances are corrected and adequate respiratory support is provided. The patient is still being rewarmed, volemic deficits are corrected by transfusion and other substitutes. Hypo–coagulability is corrected and acid–base equilibrium is restored. In this stage, the injury score is corrected and completed.

The third and final stage is represented by the complete repair of all the injuries while trying to prevent or minimize the complications.

These classic three stages have been reconsidered and, today we can identify 5 stages in damage–control approach (Table 1). This five–stage approach highlights the difference for when a patient is eligible for damage–control, and when the decision is made to resort to damage–control. Also, it is evident that frequently when applying damage–control, the need for the ‘relook’ in the abdomen and definitive surgical procedure is not entirely superposable with definitive closure of the abdomen which is one of the most problematic stages of this concept.

**Table 1:** The evolution of the damage–control concept

Stage		Stage	
1	Patient selection and damage–control	1	Patient selection
		2	Damage–control
2	Recovering towards normal physiology in ICU	3	Recovering towards normal physiology in ICU
3	Final surgical procedure and definitive closure of the abdomen	4	Relook or definitive surgical procedure
		5	Definitive closure of the abdomen

The most important factors which prompt the surgeon to switch to abbreviated laparotomy are considered to be (Moore et al.) [6]:

- Inability to achieve haemostasis owing to a recalcitrant coagulopathy.
- Inaccessible major venous injury (e.g., retrohepatic vena cava, pelvic veins)
- Time–consuming procedure in the patient with suboptimal response to resuscitation (e.g., pancreaticoduodenectomy, complex vascular reconstruction)
- Management of extraabdominal life–threatening injury (e.g., active pelvic haemorrhage necessitating angiography, torn thoracic aorta)
- Reassessment of intraabdominal contents (e.g., compromised intestinal blood supply due to extensive mesenteric injuries)
- Inability to reapproximate abdominal fascia due to splanchnic reperfusion–induced visceral oedema (e.g., following protracted shock that requires massive fluid administration)

Finding objective criteria for resorting to abbreviated laparotomy is difficult. Some parameters have been used, but there is no unanimous point of view. Most important parameters are:

- PT or PTT twice the normal (measured while in surgery)
- Massive transfusions in a short time (within 4 hrs) – over 10 units of blood
- Severe cellular shock defined by index of oxygen consumption (VO2I) < 110 ml/min/m2
- Lactacidemia of over 5 mmol/l
- pH<7,2
- Base deficit (BE)>14
- Hypothermia<34 C

Still, there is no definitive standard to allow objective evaluation when abbreviated laparotomy is necessary, as different authors have different criteria [5,7,8]. As such, the above–mentioned criteria, while representative, are not the only ones being used. Other authors have accentuated the surgeon's role in choosing the moment to stop the procedure [4]. While trying to create a standard for damage–control procedure, Asensio et al. have retrospectively analyzed 546 patients in a period of over 6 years. Parameters validated by this study are shown below [7] in Table 2.

**Table 2:** Predictive parameters for abbreviated laparotomy

Hypothermia≤34
pH≤7,2
Serum bicarbonate≤15mEq/l
Transfusion≥4000 ml blood
Transfusion≥p 5000 ml of blood and derivatives
Volemic substitute≥12000 ml while in surgery
Clinical aspects of hypocoagulability

A more holistic approach for considering damage–control surgery can further divide some strong predictors for this concept in preoperative and intraoperative (Table 3) [8].

**Table 3:** Criteria for recognising the need for damage–control (after 6)

*Preoperative*
Multiple mass casualties
Multisystem trauma with major abdominal injury
Open pelvic fracture with major abdominal injury
Major abdominal injury with need to evaluate early possible extraabdominal injury
Traumatic amputation of a limb with major abdominal injury
Need for emergency department thoracotomy
Presence of sustained hypotension (<90 mm Hg)
Presence of coagulopathy
Presence of hypothermia
Need for the adjunctive use of angioembolization
*During surgery*
Need for intraoperative thoracotomy
Major abdominal vascular injuries
Major thoracic vascular injuries
Severe complex hepatic injuries
Presence of bowel oedema/ischemia

The most important message of this paper could be that the decision to resort to damage–control, and implicitly the necessity for abbreviated laparotomy is only partially quantifiable. Abbreviated laparotomy must be chosen before the lethal triad sets in. Delaying this decision makes any approach useless, including damage–control. Damage–control is not desperate surgery, but a physiological approach to a critical (traumatic) situation. Paradoxically the more serious the patient's status is the less is undertaken from a surgical point of view. From damage–control surgery point of view, this means temporary hemorrhagic control, contamination control and temporary closing the abdomen. The speed factor is essential here.

The patient is set on his back, and the sterile field is readied from thighs to cervical area. The great median laparotomy is recommended for a good exposure. Sometimes, extending the incision to a thoracic level is accomplished by excising the xiphoid or median sternotomy.

Once decided, either in the preoperative period or during surgery, damage–control surgery will be applied. At first temporary packing will be performed to locate hemorrhagic sources. As such, a quick inspection of the four quadrants where hemostasis fields have been placed will be performed. For visible sources of bleeding, definitive hemostasis is accomplished if possible. For hepatic lesions, the injury type is decisive for selecting the way temporary hemostasis is performed.

 It is of utmost importance to note that while surgical maneuvers are being performed, the patient is being resuscitated. Extra care is taken to rewarm and fulfill the volemic needs of the patient. For rewarming the patient different methods are used (external electric devices, administrating heated fluids–blood and derivates included–and even peritoneal lavage with warm saline). Arterial–venous shunts can also be used [4,9].

For major abdominal vessels (mesenteric artery, iliac arteries) installing temporary shunts when suturing them is not possible fits within the damage–control concept. Temporary shunts are relatively easy to use by inserting catheters (most frequently Pruitt or Inahara) between the vascular ends.

Contamination control of gastro–intestinal spillage implies temporary suture of intestinal ends with mechanical staplers thus dramatically reducing the time. No anastomosis will be attempted at this point.

Coming back to hepatic packing, as Moore et al [6] propose, after the first packing rebalancing the patient whilst on the operating table is continued for another 30 minutes. We then move on to re–examining the abdomen by progressively removing the packing fields. Re–examining can show damage that has initially been missed. The importance of this step is very clear. The medical consequences can be grave if this is omitted. If bleeding control is established, the abdomen can be permanently closed. Abdominal packing may also be used for controlling bleeding from massive pelvic lesions.

For patients which require damage–control, the presence of splenic injuries implies emergency splenectomy. Any attempt of preserving the spleen in this context is perilous.

Usually, the visceral edema constantly present in these patients as well as the packing of different regions makes closing the abdomen impossible. This is why temporary closure is required. It can be accomplished in many ways, either by rapidly closing the tegument (towel clips) but this is still risky, or more likely by means of Bogota–bag method or various vacuum packs, meshes, each with their own specific advantages and disadvantages. These methods allow the avoidance of compartment syndrome, a frequent complication of severe trauma, and also make the final procedure easier to accomplish. Due to the similar results of various methods, we prefer the Bogota–bag method and, especially the vacuum–pack for its low cost and ease of usage. It was introduced over 30 years ago by a Columbian surgeon (G. Landoni) as a temporary abdomen–closing method. It is accomplished by using a sterile urinary bag, tailored on the spot to the dimensions of the parietal defect. It is then sutured with a continuous suture to the edges of the skin, and not to the fascia to preserve as much of the latter as possible for the definitive procedure. There are other options already mentioned, such as the vacuum pack, which entails placing a sheet over the intestines, then 2 sterile fields and 2 drains with a little suction, over which an adhesive foil is placed. Others use absorbable meshes or PTFE but without notable advantages, at a greatly increased cost and with an increased likelihood of intestinal fistula. Generally, the fluid losses of an open abdomen can be quite large and, with some of the mentioned methods difficult to control. Volemic resuscitation must take them into account. It seems that vacuum–pack is the most acceptable method used in practice.

The definitive procedure will be performed 36–48–72 hours after the patient has been treated in the ICU. During this time, hypothermia, hipocoagulability and acidosis are corrected, and the respiratory status must be well addressed. The goal is to restore the proper physiological balance. Sometimes uncontrolled bleeding prompts the second procedure, but in general, the purpose of the programmed second intervention is to finalise repairing of vital organs and, if possible closing definitively the abdominal wall, which is not always the case.

Using damage–control surgery has increased the survival rate for previously low-chance polytrauma patients up to 50–70%. This increase has not been without its price. Higher morbidity is to be accepted due to an increase in multiple system organ failures, septic complications (abdominal abscesses) and abdominal wall problems (incisional hernia, wound dehiscence).In the end, we can conclude that:

- Damage–control surgery is a psysiological and not anatomical approach to a major problem–the severe polytrauma.
- Damage–control surgery is not desperate surgery, even if it saves the patient life. The decision to perform damage–control must be taken before the patient is irreversibly degraded.
- Intensive care is an important stage of damage–control and it's result depends heavily on the correction of the above–mentioned disturbances.
- Damage–control approach is not risk–free, but offers the chance of survival in critical situations, as long as the decision to act is made before the patient's resources are exhausted.
